# Functional data analysis of college students’ sleep patterns and their relationships with academic performance and social networks: A four-year longitudinal study

**DOI:** 10.1371/journal.pone.0351120

**Published:** 2026-07-01

**Authors:** Yao Zhao, Haoyu Zhou

**Affiliations:** 1 Department of Statistics, Operations, and Data Science, Temple University, Philadelphia, Pennsylvania, United States of America; 2 Department of Epidemiology and Biostatistics, Temple University, Philadelphia, Pennsylvania, United States of America; Shandong University, CHINA

## Abstract

**Background:**

College students are subject to insufficient sleep and irregular sleep patterns. Most existing studies regarding sleep behaviors have relied on static measures or discrete time points for analyzing sleep data, potentially missing the dynamic and continuous nature of sleep behavior.

**Objectives:**

To examine sleep pattern evolution and their relationships with academic performance and social networks among college students using functional data analysis.

**Methods:**

This study introduces functional data analysis to examine sleep patterns and their relationships with academic performance and social networks among college students throughout their four-year undergraduate experience. Using data from the NetHealth Project, we analyzed daily sleep records from Fitbit devices worn by 76 undergraduate students, along with their academic records and social network data, comprising 61,225 daily observations. We employed functional regression to model time-varying relationships between sleep and GPA, and functional t-tests to compare sleep patterns between students with different social activity levels.

**Results:**

Sleep duration increased significantly across the undergraduate years, with pronounced seasonal fluctuations corresponding to academic cycles. The relationship between sleep and academic performance remained consistently positive throughout college, with each GPA point associated with 27.4 additional minutes of sleep on average. This relationship exhibited a U-shaped temporal pattern, strongest during freshman year (54 minutes/GPA point), weakest during junior year (5 minutes/GPA point), and recovering during senior year (48 minutes/GPA point). Social network characteristics showed no statistically significant associations with sleep patterns, though students with larger networks consistently slept slightly less than those with smaller networks.

**Conclusions:**

This study demonstrates the utility of functional data analysis in sleep research, revealing dynamic patterns in sleep behavior and time-varying relationships with academic performance that traditional discrete-time analyses would not capture. The consistently positive association between sleep duration and academic performance was maintained throughout the four-year undergraduate experience, with temporal variations suggesting the relationship is strongest during freshman and senior years. These findings provide longitudinal evidence about sleep patterns and their correlates among college students, with potential implications for the timing of sleep-related support services.

## Introduction

### Background

Sleep is a fundamental aspect of human health that plays a crucial role in cognitive function, academic performance, and social interactions [1–3]. Research has established several key mechanisms through which sleep affects academic outcomes, with memory consolidation being one of the most essential pathways [4]. Studies among college students have demonstrated that better sleep quality is associated with improved overall well-being and academic functioning [5,6], whereas chronic sleep deprivation predicts not only lower grade point averages but also a reduced likelihood of obtaining a college degree [6]. Besides these chronic effects, a study that conducted two meta-analyses found that sleep deprivation both before and after learning leads to poorer retention of the newly acquired learning material. This detrimental effect, although varying in magnitude, has been observed for both procedural memory tasks and declarative memory tasks across orginal studies [4].

College students are widely recognized as a population particularly vulnerable to insufficient or irregular sleep [7–9]. The prevalence of inadequate sleep among college students ranges from 42% to 76% across universities [6,8,9]. Among nursing students specifically, research shows that nearly three-fourths (73%) can be classified as poor sleepers [10]. The transition to college life, high academic demands, and expanding social networks all contribute to changed sleep hours and often challenged sleep patterns [11–14]. Alongside other common factors such as emotional intelligence that are critical for coping with academic stress [15], poor sleep quality among students is associated with both decreased academic performance and reduced overall well-being, including greater daytime dysfunction and compromised physical and mental health. Additionally, college students experience a higher prevalence of daytime sleep (50%) compared with other adults (36%) [16,17].

## Gaps in prior research

Despite the importance of understanding sleep patterns among college students, existing research has three primary limitations. First, most studies have relied on cross-sectional designs or short-term longitudinal analyses spanning only one semester or academic year [9]. These study designs have limited capacity to capture the continuous evolution of sleep behavior throughout the undergraduate experience or distinguish between transient fluctuations and persistent trends. Understanding how sleep patterns develop and change across the entire college career requires extended longitudinal observation that few studies have undertaken.

Second, many studies depend on self-reported sleep measures, which may be less precise than objective measurements [8,18]. Self-report surveys rely on participants’ memory and perception of their sleep, which may differ substantially from objective reality. The emergence of consumer-grade wearable devices such as Fitbit has enabled continuous, objective monitoring of sleep patterns in naturalistic settings [19]. These devices provide detailed, time-stamped data on sleep duration, timing, and quality without the burden of daily self-reporting or the expense and intrusiveness of laboratory polysomnography [20,21].

Third, traditional analytical approaches have treated sleep as a static characteristic measured at discrete time points, such as average sleep duration per semester or sleep quality assessed at a few survey occasions. For example, a traditional analysis might calculate that a student averaged 420 minutes of sleep in fall semester and 425 minutes in spring semester. These two summary statistics would completely obscure whether sleep remained stable throughout each semester or deteriorated progressively from 450 minutes in early September to 390 minutes during December finals, a pattern that has very different implications for student well-being and for the timing of interventions. Similarly, traditional regression methods typically estimate a single coefficient representing the sleep-GPA relationship (e.g., β=25 minutes per GPA point), assuming this association remains constant across all four college years. Such approaches cannot detect whether the sleep–GPA relationship is strongest during the stressful freshman transition versus later years when students have developed diverse coping strategies, or how this relationship may vary in strength over time. Analytical methods that can model sleep as a continuous, evolving process and capture time-varying relationships are needed to fully understand these complex dynamics.

Understanding the dynamic interplay between sleep, academic performance, and social networks is particularly important during college, when students face competing demands and must balance academic responsibilities, social engagement, and health behaviors. These relationships are unlikely to remain constant, as academic pressure varies across semesters, social networks evolve, and sleep patterns both influence and are influenced by these changes. Identifying *when* during college these relationships are strongest can inform intervention timing. The continuous nature of wearable device data provides an opportunity to examine these dynamics at high temporal resolution, but traditional analytical approaches that aggregate data across time periods cannot capture this evolution. This motivates the application of functional data analysis, which models sleep as a continuous trajectory and allows relationships with academic and social factors to vary over time.

While functional data analysis has been successfully applied in various research domains such as brain connectivity analysis and gene expression studies [22,23], its application to sleep research remains limited. To our knowledge, no prior studies have applied FDA to examine how sleep patterns evolve continuously throughout the multi-year college experience or to model time-varying relationships between sleep and academic outcomes using objective wearable device data. The availability of continuous Fitbit monitoring data from the NetHealth Project, spanning four years with daily sleep measurements, provides an opportunity to apply FDA methods to capture the dynamic nature of college students’ sleep behavior, including seasonal fluctuations, gradual trends, and evolving relationships with academic and social factors that traditional discrete-time approaches cannot adequately address. This study makes primarily an empirical contribution by revealing these dynamic patterns, while methodologically demonstrating FDA as an enabling tool particularly well-suited for examining continuous temporal processes in naturalistic settings.

## Study objectives

The objective of this study is to conduct a longitudinal examination of sleep patterns across the four-year undergraduate experience and to investigate their relationships with academic performance and social network characteristics over time.

To further address this objective, we consider the following research questions

**Research question 1:** How do sleep patterns evolve throughout the four-year college experience?

*Hypothesis 1:* We hypothesize that sleep patterns will show seasonal fluctuations corresponding to academic cycles, with gradual increases in sleep duration as students progress through college years.

**Research question 2:** What is the time-varying relationship between sleep patterns and academic performance?

*Hypothesis 2:* We hypothesize that better sleep will be consistently associated with higher academic performance, but this relationship will vary in strength across the four-year college experience.

**Research question 3:** How do social network characteristics relate to sleep patterns throughout college?

*Hypothesis 3:* We hypothesize that students with different levels of social activity will exhibit distinct sleep patterns, with those having larger social networks and stronger relationships potentially sleeping less due to time devoted to social engagement.

Our findings can inform the development of targeted interventions and support services for improving students’ sleep habits, ultimately contributing to better academic outcomes and overall college experiences.

## Methods

### Data source and study design

This study uses data from the NetHealth Project, a comprehensive longitudinal investigation of social networks, health behaviors, and academic outcomes among college students [24]. The project was conducted at the University of Notre Dame from 2015 to 2019 and funded by the National Health, Lung and Blood Institute of the National Institutes of Health (5R01HL117757−04). The primary objectives of the NetHealth Project were to examine how social network structures influence health behaviors and outcomes among college students, with particular emphasis on the interplay between social connections, physical activity, sleep patterns, and psychological well-being throughout the undergraduate experience.

The project recruited 617 first-year undergraduate students who entered Notre Dame as freshmen in Fall 2015. Participants were initially enrolled for a two-year study, and 300 students continued participation in this study for additional two years. Data collection concluded in May 2019, and the dataset is now publicly available through the NetHealth Project repository at the University of Notre Dame’s Center for Network Science and Data. The authors obtained access to this de-identified dataset through the public repository for secondary analysis.

Each participant was provided with a Fitbit device (Fitbit Charge HR or Charge 2, depending on enrollment wave) to continuously track their sleep patterns and physical activity throughout their undergraduate years. The Fitbit devices were synchronized with the Fitbit cloud server, from which data was retrieved via application programming interface (API). The sleep data collected included daily sleep duration, sleep onset and wake times, and sleep interruptions. Additionally, participants completed periodic surveys assessing social network characteristics, academic experiences, and health behaviors at eight waves throughout the study period (Summer 2015 through Spring 2019). For a subset of participants who provided consent, academic performance data were obtained from the University of Notre Dame Registrar’s Office, including course enrollments and letter grades for each semester.

The longitudinal design of the NetHealth Project is particularly well-suited for our research questions. Preliminary descriptive analyses of the NetHealth data revealed substantial variability in sleep patterns both within and across students, with clear seasonal patterns corresponding to academic calendars. These patterns, combined with the availability of objective sleep measurements over the entire undergraduate experience and concurrent academic performance data, suggested that traditional discrete-time analytical approaches might fail to capture important temporal dynamics. The continuous nature of the Fitbit data collection and the extended four-year observation period provide an ideal opportunity to apply functional data analysis methods to examine how sleep patterns evolve over time and how they relate to academic and social factors in ways that cross-sectional or short-term studies cannot address.

Attrition occurred primarily at the transition from the initial two-year study to the extended four-year follow-up, when participants were asked to re-consent for continued participation. Additional attrition occurred due to students transferring to other institutions, withdrawing from the university, or discontinuing study participation for personal reasons. The study protocol was approved by the Institutional Review Board at the University of Notre Dame, and all participants provided informed consent.

### Variable definitions and operationalization

**Sleep Duration.** Daily sleep duration was measured using Fitbit devices that participants wore continuously throughout the study. The variable *minsasleep* represents total minutes asleep per night, recorded automatically by the Fitbit’s accelerometer-based sleep detection algorithm. When multiple sleep episodes occurred within a single day, we summed these to obtain total daily sleep duration. Multilpe studies have indicated that Fitbit wearable devices provide generally acceptable performance not only for measuring sleep duration but also for identifying sleep stages, and these devices have been applied in studies to measure sleep patterns [25–28].

**Academic Performance.** Academic performance was measured using semester GPA calculated from official course grade records. Letter grades were converted to numeric values on a standard 4.0 scale (A=4.000, A−=3.667, B+=3.333, etc.). Pass/fail or non-graded courses (e.g., S for Satisfactory, U for Unsatisfactory, P for Pass, W for Withdrawal, and NR for No Report) were excluded from GPA calculations. For the functional regression analysis, GPA was represented as a step function that remained constant within each semester and changed at semester boundaries.

**Social Network Size.** Social network size was operationalized as the number of unique social contacts (*alterid*) reported by each participant in the Network Survey. Participants completed surveys at eight waves throughout their undergraduate years (Summer 2015 through Spring 2019), naming up to 25 individuals with whom they had regular contact [24]. This upper limit was defined by the survey design to balance comprehensive network reporting with respondent burden and ensure consistency across participants. For each student, we calculated the number of contacts per wave and then averaged across all completed waves to obtain a stable measure of network size. The mean network size was 12.9 contacts per student (SD = 5.2). Students were classified into high and low social activity groups using a median split (11.6 contacts) of this average network size.

**Relationship Strength.** Relationship strength was operationalized as a composite measure combining frequency of interaction and relationship closeness. Frequency of interaction (*freq*) was assessed with the question “On average, how frequently did you interact with each person during the last 3 months?” with response options: Daily, Weekly, Monthly, or Less Often. Relationship closeness (*close*) was assessed by asking participants whether each contact was “especially close” (one of their closest personal contacts), “merely close” (enjoyable but not among closest contacts), “less than close” (no wish to develop friendship), or “distant” (do not enjoy spending time unless necessary). We created a composite relationship strength score by assigning numeric values to each response (Frequency: Daily = 3, Weekly = 2, Monthly = 1, Less Often = 0; Closeness: Especially Close = 3, Merely Close = 2, Less Than Close = 1, Distant = 0) and summing these scores for each reported contact. Total strength scores were calculated per student per wave and averaged across waves. Students were classified into high and low relationship strength groups using a median split.

### Missing data handling and participant inclusion

Missing daily observations were inevitable due to Fitbit device non-wear, battery depletion, synchronization failures, or technical malfunctions. We did not impute missing values. Instead, we leveraged the inherent capability of functional data analysis to handle irregularly spaced observations: the B-spline smoothing procedure fits continuous curves to available data points, effectively interpolating across gaps while preserving the temporal structure of observed sleep patterns. This approach is preferable to explicit imputation methods for functional data as it avoids introducing artificial data points while still producing smooth continuous functions.

To ensure reliable functional curve estimation, we required participants to have sleep data for at least 50% of the study period (678 of 1,356 possible days from August 26, 2015 to May 13, 2019). This threshold balances sample retention with data quality, as B-spline smoothing requires sufficient temporal coverage to produce stable estimates. Additionally, participants were required to have academic records (GPA data) for linkage with sleep patterns and have at least one wave of the Network survey. Applying these criteria resulted in a final analytical sample of 76 students with 61,225 daily sleep observations.

Because inclusion required sufficient Fitbit wear-time, availability of GPA records, and at least one completed Network Survey, the final analytic sample represents a more compliant subset of the original cohort. This may limit generalizability if more compliant students differ systematically in sleep behavior, academic performance, or social network characteristics from those who attrited or declined additional consent. However, these potential biases are unlikely to affect the internal validity of our findings regarding relationships between sleep patterns and academic performance within our analytical sample.

### Analysis methods

We applied functional data analysis (FDA) to model each student’s daily sleep duration as a continuous function over time rather than discrete observations. This approach was chosen because sleep patterns exhibit inherent continuity and temporal dependencies that traditional static analyses cannot capture, allowing us to preserve the temporal structure of sleep data and examine dynamic relationships with academic and social factors.

To convert discrete daily sleep measurements into smooth functions, we employed cubic B-spline basis functions (order 4), chosen for their optimal balance between flexibility and smoothness for longitudinal sleep data. The number of basis functions was determined using generalized cross-validation (GCV), which consistently indicated 15–20 basis functions as optimal for capturing both seasonal patterns and day-to-day variations without overfitting. Knots were placed at equally spaced intervals throughout the four-year study period, with additional knots positioned at semester boundaries to better capture academic cycle transitions. This strategic placement allows the model to adapt to known structural changes in students’ routines while maintaining smoothness within academic periods.

For examining time-varying relationships between sleep patterns and academic performance, we employed concurrent functional linear regression. Time-varying coefficients were estimated using roughness penalty methods with smoothing parameters selected via restricted maximum likelihood, balancing model fit with smoothness to prevent overfitting while capturing genuine temporal variations.

To analyze differences in sleep patterns between students with varying social activity levels, we implemented pointwise functional t-tests comparing mean sleep functions between groups at each time point. Students were classified into high and low social activity groups using median splits of self-reported close contacts and relationship intimacy scores, calculated separately for each academic year to account for temporal changes in social network formation. The choice of pointwise testing was motivated by our specific interest in identifying when during college social activity levels most strongly influence sleep patterns. To address multiple testing across time points, we used the maximum statistic approach with permutation testing (1000 permutations), providing strong control of family-wise error rate while maintaining reasonable power for detecting differences during specific academic periods.

All analyses were conducted in R version 4.3.0 using the fda package for functional data analysis and refund package for functional regression models. We conducted sensitivity analyses to validate our methodological choices, including alternative smoothing parameter selection using AIC and BIC criteria, different numbers of basis functions (10, 15, 20, 25), comparison with traditional mixed-effects models, and bootstrap confidence intervals for functional regression coefficients. These analyses confirmed the robustness of our main findings across different analytical approaches.

### Ethics statement

The original NetHealth Project data collection was approved by the Institutional Review Board at the University of Notre Dame, and all participants provided informed consent. The present study involves only secondary data analysis of de-identified NetHealth data and was conducted in accordance with Temple University human subjects research policies.

## Results

Table 1 presents baseline characteristics of the 76 students included in the analytical sample, comprising 61,225 daily sleep observations. The sample was relatively balanced by gender (39 female, 37 male). Mean baseline sleep duration was 430 minutes (SD = 38.6) for females and 417 minutes (SD = 34.6) for males (*p* = 0.11). Baseline GPA was similar between groups (females: 3.63, SD = 0.25; males: 3.72, SD = 0.24; *p* = 0.107). Social activity measures did not differ by gender, with approximately half of each group classified as high activity. Significant gender differences were observed in academic major distribution (*p* = 0.012), with females more likely to major in Science (48.7% vs 18.9%) and males more likely to major in Engineering (51.4% vs 17.9%). The majority of students were from the East North Central region (43.4%) and had parents living together (86.8%).

**Table 1:** Baseline characteristics of the analytical sample by gender (N = 76).

	Female (N = 39)	Male (N = 37)	P value
**Average sleep duration (mins)**			
Mean (SD)	430 (38.6)	417 (34.6)	0.11
Median [Min, Max]	438 [314, 481]	417 [341, 492]	
**Baseline GPA**			
Mean (SD)	3.63 (0.249)	3.72 (0.241)	0.107
Median [Min, Max]	3.71 [3.07, 4.00]	3.78 [3.11, 4.00]	
**Activity Group**			
High	21 (53.8%)	20 (54.1%)	1
Low	18 (46.2%)	17 (45.9%)	
**Relationship Strength Group**			
High	21 (53.8%)	19 (51.4%)	1
Low	18 (46.2%)	18 (48.6%)	
**Major type**			
Arts & Letters	6 (15.4%)	4 (10.8%)	0.0118
Architecture	1 (2.6%)	1 (2.7%)	
Engineering	7 (17.9%)	19 (51.4%)	
Business	5 (12.8%)	6 (16.2%)	
Science	19 (48.7%)	7 (18.9%)	
Missing	1 (2.6%)	0 (0%)	
**Region in the US**			
East North Central	17 (43.6%)	16 (43.2%)	0.856
East South Central	1 (2.6%)	2 (5.4%)	
Middle Atlantic	4 (10.3%)	4 (10.8%)	
Mountain	2 (5.1%)	1 (2.7%)	
New England	1 (2.6%)	0 (0%)	
Pacific	3 (7.7%)	1 (2.7%)	
South Atlantic	2 (5.1%)	5 (13.5%)	
West North Central	4 (10.3%)	4 (10.8%)	
West South Central	2 (5.1%)	4 (10.8%)	
Missing	3 (7.7%)	0 (0%)	
**Parent Status**			
Divorced or living apart	8 (20.5%)	2 (5.4%)	0.0872
Living together	31 (79.5%)	35 (94.6%)	

Note: P-values were obtained through statistical tests comparing covariates between males and females. Welch’s t-tests were used for continuous covariates, $\chi^{2}$ tests for categorical covariates, and Fisher’s exact tests for categorical covariates with small counts (<5).

**Fig 1:**
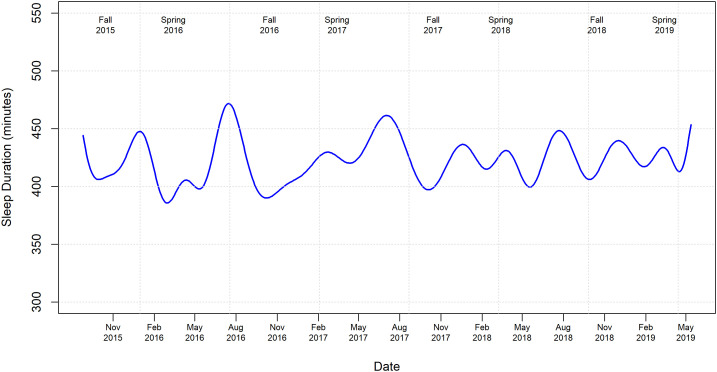
Sleep pattern of a representative student throughout their four-year undergraduate study (2015-2019). The y-axis shows daily sleep duration in minutes, and the x-axis shows the timeline with month and year labels. The smoothed curve was estimated using B-spline basis functions. Notable dips in sleep duration coincide with high-workload academic periods, particularly during fall semester finals (November-December) and spring semester examinations (February-May), while peaks occur during summer and winter breaks.

Fig 1 illustrates the sleep pattern of a representative student throughout their four-year undergraduate study (2015–2019). The smoothed functional curve reveals several notable patterns in sleep behavior. The figure demonstrates consistent cyclical patterns in sleep duration corresponding to academic cycles. Sleep duration peaks are visible during summer break periods (July-August), while troughs occur during the academic semesters. Particularly pronounced dips in sleep duration are observed during high-workload periods, including fall semester finals (November-December) and spring semester midterms and finals (February-March and April-May). Furthermore, within each semester, sleep duration typically decreases from the beginning to the end of the term, reflecting increasing academic demands as courses progress toward examinations. Recovery periods are evident during semester breaks, when sleep duration increases substantially. Last, examining the overall trajectory across the four years, the student’s sleep pattern shows relative stability in the general range of fluctuation (approximately 380–470 minutes). The amplitude of fluctuations appears somewhat reduced in later years (junior and senior).

**Fig 2:**
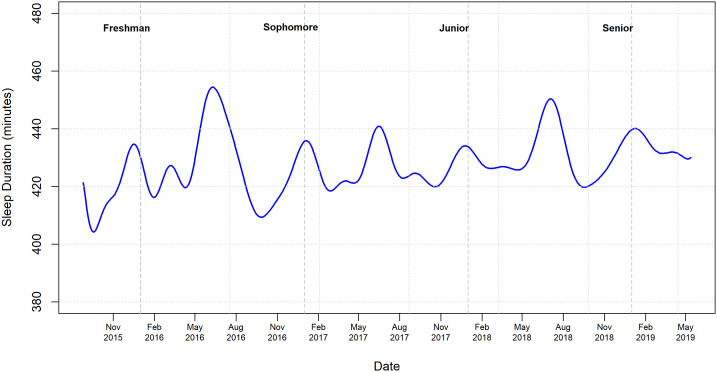
Average sleep duration across 76 students from freshman year (2015) to senior year (2019). The *y*-axis shows daily sleep duration in minutes, and the *x*-axis shows the timeline. The smoothed curve was estimated using B-spline basis functions applied to the daily mean sleep duration across all students. A general upward trend is observed from freshman to senior year, with periodic fluctuations corresponding to academic cycles. Peak sleep durations occur during summer breaks, while troughs appear during high-workload academic periods.

Fig 2 presents the average sleep duration across all 76 students throughout their four-year undergraduate experience (2015−2019). The smoothed functional curve reveals both consistent seasonal patterns and a long-term trend in sleep behavior. Similar to individual patterns, the average sleep duration exhibits pronounced cyclical fluctuations corresponding to academic cycles. Peak sleep durations occur during summer periods (May-August), with the highest peaks observed in Summer 2016 (455 minutes) and Summer 2018 (450 minutes). Troughs consistently appear at the beginning of fall semesters (August-September) and during high-stress academic periods (November and February-March). Furthermore, we find the general upward trend in average sleep duration from freshman to senior year. During the freshman year (2015−2016), the average sleep duration typically fluctuated around 410−430 minutes (6.8–7.2 hours), while by senior year (2018−2019), students averaged approximately 420−440 minutes (7.0–7.3 hours) of sleep per night. This gradual increase was statistically significant (β=4.13 minutes/year, 95% CI: [2.26, 6.00], *p* < 0.001). The patterns observed in Fig 2 (population average) closely mirror those seen in Fig 1 (individual student), confirming that the seasonal fluctuations and long-term trends are consistent across the student population rather than being idiosyncratic to particular individuals.

**Fig 3:**
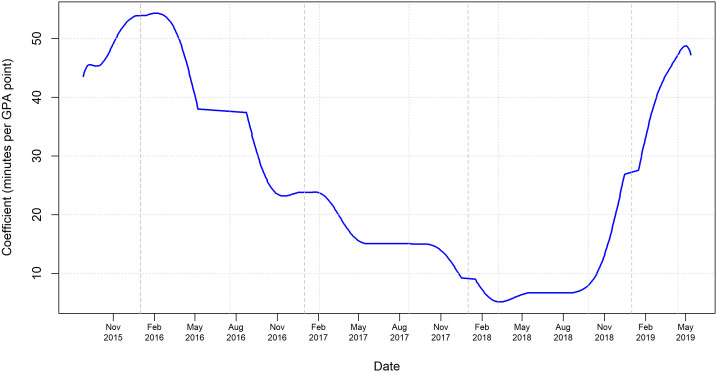
Time-varying coefficients from functional regression analysis showing the relationship between GPA and sleep duration throughout the undergraduate years. Positive coefficients indicate that higher GPA is associated with longer sleep duration. The coefficient remains positive throughout the entire study period (100%), with a U-shaped pattern: strongest during freshman year (peak 54 minutes per GPA point), declining through sophomore and junior years (minimum 5 minutes), and recovering during senior year (48 minutes).

**Fig 4:**
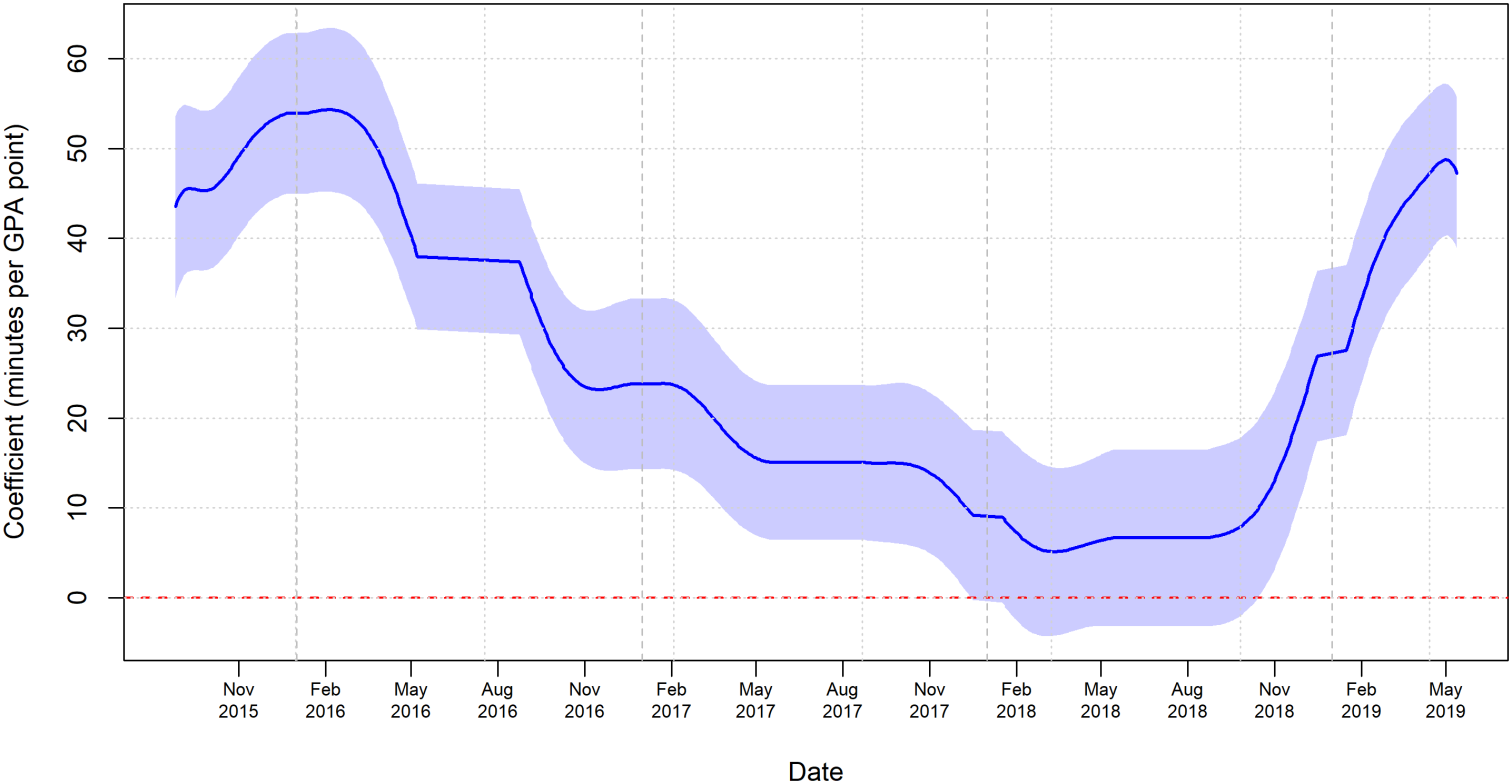
Time-varying coefficients from functional regression analysis with 95% confidence intervals. The blue line represents the estimated coefficient, while the shaded region indicates uncertainty. The red dashed line indicates zero. The confidence interval excludes zero for 81.6% of the study period, indicating statistically significant positive associations between GPA and sleep duration during most of the undergraduate experience.

We then employ functional regression analysis to investigate the relationship between sleep duration and academic performance (GPA). Figs 3 and 4 present the time-varying coefficients from the analysis, illustrating how sleep duration impacts academic performance over time. The coefficient remained positive throughout the entire study period (100% of time points), with 81.6% of time points showing statistically significant positive associations (95% CI excluding zero). On average, each one-point increase in GPA was associated with 27.4 additional minutes of sleep per night (95% CI: 12.3 to 42.5 minutes).

The strength of this relationship exhibited a notable U-shaped pattern across the four years. The association was strongest during freshman year, peaking at approximately 54 minutes per GPA point during Spring 2016. The relationship then declined progressively through sophomore and junior years, reaching its minimum of approximately 5 minutes per GPA point during Spring 2018. Notably, the relationship strengthened again during senior year, returning to approximately 48 minutes per GPA point by Spring 2019.

Functional t-tests with permutation testing (1000 permutations) compared sleep patterns between students classified into high versus low social activity groups based on two measures: network size (number of social contacts) and relationship strength (composite score of interaction frequency and closeness). Neither comparison revealed statistically significant differences in sleep patterns at any point during the undergraduate years. For network size, students in the high activity group (*n* = 41) averaged 417.5 minutes (6.96 hours) of sleep compared to 430.1 minutes (7.17 hours) for the low activity group (*n* = 35), a difference of 12.6 minutes (maximum t-statistic = 1.93, critical value = 2.84, *p* > 0.05). For relationship strength, students with high scores (*n* = 40) averaged 419.6 minutes (6.99 hours) compared to 427.4 minutes (7.12 hours) for those with low scores (*n* = 36), a difference of 7.9 minutes (maximum t-statistic = 1.68, critical value = 2.85, *p* > 0.05). Although these differences did not reach statistical significance, a consistent pattern emerged: students with more extensive social networks and stronger social relationships tended to sleep slightly less than their peers with smaller networks.

### Demographic associations with sleep and academic performance

We also examined whether sleep patterns and their relationships with academic performance varied by demographic characteristics.

**Gender.** Gender-stratified functional regression analyses revealed differences in the magnitude of the sleep-GPA relationship between females and males. The average time-varying coefficient was 41.8 minutes per GPA point for females (95% CI: [28.7, 54.8]) and 22.6 minutes per GPA point for males (95% CI: [10.7, 34.6]). While both genders showed positive sleep-GPA relationships throughout college, the association was approximately 19 minutes stronger for females. However, confidence intervals overlapped at 47.8% of timepoints, indicating that this difference varied over time and was not consistently significant across all academic periods.

**Academic Major.** Academic major distribution differed significantly by gender (*p* = 0.012), with more females majoring in Science (48.7%) and more males in Engineering (51.4%). However, academic major did not significantly influence average sleep duration (*F* = 0.12, *p* = 0.726). Major-stratified functional regression revealed similar sleep-GPA relationships for STEM majors (29.4 minutes/GPA point) and non-STEM majors (26.4 minutes/GPA point), with a difference of only 2.9 minutes.

Given the minimal differences by major type and the time-varying nature of gender differences, we did not include demographic covariates in the primary functional regression models. The consistently positive sleep-GPA associations across both genders and all major types support the robustness of our primary findings.

## Discussion

### Summary of key findings

This study applied functional data analysis to examine sleep patterns and their relationships with academic performance and social networks among 76 college students throughout their four-year undergraduate experience. Our primary contribution is empirical: revealing dynamic patterns in sleep behavior and its associations with academic and social factors that traditional discrete-time analyses would not capture.

Three key findings emerged from our analyses. First, sleep duration increased significantly across the undergraduate years (β = 4.13 minutes/year, *p* < 0.001), with pronounced seasonal fluctuations corresponding to academic cycles. Students averaged 420.0 minutes of sleep during freshman year, increasing to 428.6 minutes by senior year. Second, the relationship between sleep and academic performance was consistently positive throughout college, with each GPA point associated with 27.4 additional minutes of sleep on average. This relationship exhibited a U-shaped temporal pattern, strongest during freshman year (54 minutes/GPA point), weakest during junior year (5 minutes/GPA point), and recovering during senior year (48 minutes/GPA point). Third, social network characteristics showed no statistically significant associations with sleep patterns, although students with larger networks consistently slept slightly less (12.6 minutes difference) than those with smaller networks.

### Sleep pattern evolution

Our finding aligns with existing original studies [29,30] that college students’ average sleep duration increases as they advance through the academic years, although such difference of sleep duration between students of different years may not always be statistically significant in studies [31]. The observed increase of approximately 8 minutes from freshman to senior year in this study, while modest, was statistically significant and consistent across students. This pattern may reflect improved time management skills, adaptation to academic demands, or stabilization of social routines as students progress through college. The seasonal fluctuations we observed, with sleep duration peaking during breaks and reaching nadirs during examination periods, are consistent with previous research demonstrating the strong impact of academic stress on sleep [32].

The functional approach revealed that these seasonal patterns were remarkably consistent both within individuals (Fig 1) and across the student population (Fig 2), suggesting that academic calendar structure exerts a systematic influence on student sleep behavior. Traditional analyses using semester averages would obscure these within-semester dynamics, highlighting the value of treating sleep as a continuous function over time.

### Sleep and academic performance

The consistently positive association between sleep duration and GPA throughout the undergraduate years provides longitudinal evidence supporting the importance of adequate sleep for academic success. Our finding that students with higher GPAs sleep more—not less—challenges the widespread belief [33,34] that sacrificing sleep is either necessary or beneficial for academic achievement. Similar findings surrounding this association have been documented [35,36], but our functional regression approach reveals that the strength of this relationship varies substantially across the college experiences. It is worth noting that a cross-sectional study found better academic performance among poor sleepers in Saudi Arabia [37]. Further investigation is required to explain the difference in the association direction by country.

The U-shaped temporal pattern in the sleep-GPA coefficient is a novel finding. The strong association during freshman year (54 minutes/GPA point) may reflect that during the critical transition to college, sleep habits are particularly important for academic adjustment. The attenuation during sophomore and junior years suggests that as students develop diverse coping strategies and study habits, individual differences in sleep become less tightly coupled with academic outcomes. The recovery during senior year may reflect increased academic pressure (thesis work, graduate school preparation) that re-establishes the importance of adequate sleep, or alternatively, greater self-awareness among seniors about their sleep needs.

It is important to note that our concurrent functional regression models associations, not causation. While better sleep may facilitate academic performance through enhanced memory consolidation and cognitive function [38,39], the underlying causal mechanism might also be that students with better academic skills complete work more efficiently, leaving more time for sleep. Additionally, several unmeasured factors could confound the observed relationships. Individual differences in chronotype (circadian preference), baseline cognitive ability, course workload, mental health status, caffeine and alcohol use, physical activity, part-time employment, and financial stress could all influence both sleep patterns and academic performance. While the consistency of the positive sleep-GPA association across all four years strengthens confidence in this relationship, future research incorporating comprehensive assessment of these potential confounders would help establish the independent contribution of sleep to academic outcomes.

### Gender differences in sleep-academic performance relationships

An exploratory finding from our gender-stratified analyses was that females showed a stronger average sleep-GPA relationship (41.8 minutes/GPA point) compared to males (22.6 minutes/GPA point). This suggests that adequate sleep may be more strongly associated with academic performance for female students, though the time-varying nature of this difference (CIs overlapped at nearly half of timepoints) indicates that the magnitude of gender differences fluctuates across the undergraduate years.

The literature on gender differences in sleep-academic performance relationships presents considerable heterogeneity. Our finding aligns with Marta et al. [40], who reported that insomnia was significantly associated with poor academic performance risk only in females, suggesting a female-specific vulnerability to sleep-related academic impairment. However, several other studies have reported stronger associations in males than in females. For instance, Okano et al. [14] found that sleep inconsistency was negatively correlated with academic performance in males but not in females, while Kuhn et al. [41] observed that sleep duration and quality were significant predictors of academic performance only among male students. These discrepancies across studies may reflect differences in methodological approaches (subjective versus objective sleep measures), sample characteristics (age, academic level, cultural context), or the specific sleep dimensions examined (duration, quality, consistency, or timing). The biological mechanisms underlying these gender differences remain unclear, warranting further investigation.

Importantly, our study showed that females and males share a consistently positive associations between sleep and GPA throughout college, supporting our main conclusion that adequate sleep is important for academic success regardless of gender. The gender difference we observed represents a difference in magnitude rather than direction of effect.

### Social networks and sleep

Contrary to our hypothesis, social network characteristics were not significantly associated with sleep patterns at any point during college. Although students with larger networks and stronger relationships consistently slept slightly less (7.9–12.6 minutes), these differences did not reach statistical significance. This null finding suggests that the trade-off between social engagement and sleep, while present directionally, may be too small to detect with our sample size, or that students successfully balance social activities without substantially compromising sleep.

The widening gap across academic years (from 10.3 minutes in freshman year to 15.5 minutes in senior year) suggests that social network effects on sleep may accumulate over time as students develop and maintain larger networks. Future studies with larger samples could investigate whether this trend reaches statistical significance and identify specific social behaviors (e.g., evening socializing, weekend activities) that most strongly compete with sleep time.

### Methodological contributions

While our primary contribution is empirical, this study also demonstrates the utility of functional data analysis for sleep research. FDA offers several advantages for analyzing longitudinal sleep data: it preserves the continuous, dynamic nature of sleep behavior; it accommodates irregular observation schedules common with wearable devices; and it enables modeling of time-varying relationships that traditional approaches cannot capture. The U-shaped pattern in the sleep-GPA relationship, for example, would be invisible to analyses using yearly or semester averages.

We do not claim that FDA is universally superior to other longitudinal methods such as mixed-effects models or growth curve analysis. Rather, we demonstrate that FDA is particularly well-suited for research questions about temporal dynamics and continuous trajectories in sleep behavior. Researchers should select analytical approaches based on their specific questions and data characteristics.

## Limitations

First, our analytical sample of 76 students with at least 50% data coverage represents a substantial reduction from the original NetHealth cohort (N = 617). Students maintaining high device compliance and consenting to grade release may differ systematically from those who attrited, potentially limiting generalizability. The high baseline GPAs in our sample (mean 3.67) suggest a relatively academically engaged population. It is therefore possible that the observed U-shaped pattern in the sleep–GPA association partially reflects behavioral characteristics of high-performing students, such as more effective time management or adaptive coping strategies, although this cannot be directly tested in the current study.

Second, our sample is drawn from a single selective private university, limiting generalizability to other institutional contexts. Sleep patterns and their relationships with academic and social factors may differ at public universities, community colleges, or institutions with different student demographics.

Third, our social network measures were derived from periodic surveys rather than continuous behavioral data, providing less temporal resolution than the sleep measures. More frequent assessment of social interactions could reveal dynamic relationships between daily social activities and nightly sleep.

Finally, as noted above, our correlational design cannot establish causal relationships. Experimental or quasi-experimental designs would be needed to determine whether improving sleep causes better academic performance.

### Implications and future directions

Our findings have implications for student wellness programs and academic support services. The consistent positive association between sleep and GPA suggests that interventions promoting adequate sleep may support academic success, particularly during the critical freshman transition when this association is strongest. The seasonal patterns we observed indicate that targeted sleep interventions during high-stress academic periods (midterms, finals) could be beneficial.

Future research should examine whether the patterns we observed replicate in more diverse student populations and institutional contexts. Larger samples would provide power to detect smaller effects of social network characteristics on sleep. Integration of sleep quality measures, academic engagement indicators, and more granular social interaction data could provide a more comprehensive picture of the factors influencing student sleep and its academic consequences. Finally, intervention studies testing whether sleep improvement programs enhance academic outcomes would establish the causal significance of the sleep-GPA association we observed.

## Conclusion

This study applied functional data analysis to longitudinal sleep data from 76 college students across their four-year undergraduate experience, revealing dynamic patterns in sleep behavior and its associations with academic performance. Our findings demonstrate three key patterns: sleep duration increases modestly but significantly across the college years (approximately 8 minutes from freshman to senior year), with pronounced seasonal fluctuations corresponding to the academic calendar; higher academic performance is consistently associated with longer sleep duration throughout college, though the strength of this relationship varies substantially over time following a U-shaped pattern; and social network characteristics show no statistically significant associations with sleep patterns, though students with larger networks consistently slept slightly less than their peers.

These findings contribute to understanding college student sleep behavior by documenting temporal dynamics that cross-sectional or discrete-time longitudinal analyses would not capture. The consistently positive association between sleep and academic performance provides longitudinal evidence supporting the importance of adequate sleep during the college years, particularly during the critical freshman transition when this association is strongest. However, the observational nature of our data precludes causal inference. We cannot determine whether adequate sleep promotes academic success, whether academically successful students manage time more effectively to allow for more sleep, or whether unmeasured factors such as cognitive ability, mental health, or time management skills drive both outcomes.

Our findings should be interpreted within the context of our study’s limitations. The sample is drawn from a single selective private university with high baseline academic achievement (mean GPA 3.67), limiting generalizability to other institutional contexts or student populations. The analytical sample required high device compliance and consent for grade release, potentially representing more engaged students whose sleep patterns may differ from those who did not meet inclusion criteria.

Despite these limitations, this study demonstrates that functional data analysis offers a valuable approach for examining longitudinal sleep patterns and their correlates. The method’s ability to model continuous trajectories and time-varying relationships reveals insights into the dynamic nature of sleep behavior that complement findings from traditional analytical approaches. For university stakeholders, our findings suggest that sleep may be an important factor in student academic success, particularly during the freshman year transition. Future research should examine whether these patterns replicate in more diverse student populations and institutional settings, incorporate comprehensive assessment of potential confounders, and employ experimental or quasi-experimental designs to establish causal relationships between sleep and academic outcomes.
